# Mechanisms of reduced peak oxygen consumption in subjects with uncomplicated type 2 diabetes

**DOI:** 10.1186/s12933-021-01314-6

**Published:** 2021-06-22

**Authors:** Lorenzo Nesti, Nicola Riccardo Pugliese, Paolo Sciuto, Nicolò De Biase, Matteo Mazzola, Iacopo Fabiani, Domenico Trico, Stefano Masi, Andrea Natali

**Affiliations:** 1 Metabolism, Nutrition, and Atherosclerosis Laboratory, Department of Clinical and Experimental Medicine, University of Pisa, Via Savi 10, 56126 Pisa, Italy; 2 Cardiopulmonary Laboratory, Department of Clinical and Experimental Medicine, University of Pisa, Pisa, Italy; 3 Fondazione Toscana G. Monasterio, Pisa, Italy; 4 Department of Surgical, Medical and Molecular Pathology and Critical Care Medicine, University of Pisa, Pisa, Italy

**Keywords:** Type 2 diabetes, Effort intolerance, Heart failure with preserved ejection fraction, Exercise physiology, Cardiopulmonary exercise test, Diabetic cardiomyopathy

## Abstract

**Background:**

Type 2 diabetes mellitus (T2D) increases the risk of incident heart failure (HF), whose earliest fingerprint is effort intolerance (i.e. impaired peak oxygen consumption, or VO_2peak_). In the uncomplicated T2D population, however, the prevalence of effort intolerance and the underpinning mechanistic bases are uncertain. Leveraging the multiparametric characterization allowed by imaging-cardiopulmonary exercise testing (iCPET), the aim of this study is to quantify effort intolerance in T2D and to dissect the associated cardiopulmonary alterations.

**Methods:**

Eighty-eight adults with well-controlled and uncomplicated T2D and no criteria for HF underwent a maximal iCPET with speckle tracking echocardiography, vascular and endothelial function assessment, as well as a comprehensive biohumoral characterization. Effort intolerance was defined by a VO_2peak_ below 80% of maximal predicted oxygen uptake.

**Results:**

Forty-eight patients (55%) had effort intolerance reaching a lower VO_2peak_ than T2D controls (16.5 ± 3.2 mL/min/kg, vs 21.7 ± 5.4 mL/min/kg, p < 0.0001). Despite a comparable cardiac output, patients with effort intolerance showed reduced peak peripheral oxygen extraction (11.3 ± 3.1 vs 12.7 ± 3.3 mL/dL, p = 0.002), lower VO_2_/work slope (9.9 ± 1.2 vs 11.2 ± 1.4, p < 0.0001), impaired left ventricle systolic reserve (peak S’ 13.5 ± 2.8 vs 15.2 ± 3.0, p = 0.009) and global longitudinal strain (peak-rest ΔGLS 1.7 ± 1.5 vs 2.5 ± 1.8, p = 0.03) than subjects with VO_2peak_ above 80%. Diastolic function, vascular resistance, endothelial function, biohumoral exams, right heart and pulmonary function indices did not differ between the two groups.

**Conclusions:**

Effort intolerance and reduced VO_2peak_ is a severe and highly prevalent condition in uncomplicated, otherwise asymptomatic T2D. It results from a major defect in skeletal muscle oxygen extraction coupled with a subtle myocardial systolic dysfunction.

**Supplementary Information:**

The online version contains supplementary material available at 10.1186/s12933-021-01314-6.

## Background

Defined as the inability to perform physical exercise at the maximal intensity expected (according to age, gender, body mass index (BMI), and habitual levels of physical activity [1]), effort intolerance can be quantified and objectively measured by peak oxygen consumption (VO_2peak_) during a graded maximal exercise test. Effort intolerance is the hallmark of heart failure (HF), is part of the definition of the HF syndrome and is intimately linked to its pathophysiology [2]. Given that type 2 diabetes mellitus (T2D) portends an increased risk of developing HF—especially HF with preserved ejection fraction (HFpEF)—that is neither entirely explained by traditional cardiovascular risk factors nor by coronary heart disease, an early, primary cardiopulmonary impairment has been postulated, but never clearly demonstrated [3]. Effort intolerance was previously reported in otherwise asymptomatic subjects with T2D, with slower oxygen uptake kinetics and reduced peak values in comparison to normal subjects during cardiopulmonary exercise test (CPET) [4]. Notably, reduced VO_2peak_ is a reliable predictor of cardiovascular disease, all-cause mortality [5], development of HF [6], and reduced quality of life in T2D [7]. According to current guidelines, the early appearance of effort intolerance is a marker of subclinical HF (American Heart Association stage B), whose early recognition justifies a more aggressive diagnostic-therapeutic workup. To date, however, the prevalence of effort intolerance in T2D is unclear, and so are the underlying mechanisms [4], leaving the prevention strategies for HF in T2D uncertain.

With the present study, we aim at quantifying the prevalence of effort intolerance in an outpatient, uncomplicated, and otherwise asymptomatic T2D population. Participants underwent exercise echocardiography during a maximal imaging-CPET (iCPET), which allows the dissection of the pathophysiological mechanisms underlying a reduced VO_2peak_ by simultaneous measurement of the major determinants of exercise physiology.

## Patients and methods

### Study population

We prospectively enrolled 114 patients from the Diabetes Outpatient Clinic at the Santa Chiara University Hospital of Pisa. Inclusion criteria were: men or women of 40 to 80 years of age with a clinical diagnosis of T2D according to the ADA criteria [8]; HbA_1c_ values between 53 and 69 mmol/mol (7.0 to 8.0%); on stable hypoglycemic and cardioactive therapy for at least 3 months; baseline echocardiographically-assessed left ventricle ejection fraction (LVEF) above 50%; without a dignosis of HF according to guidelines [9]. Exclusion criteria were: symptoms or diagnosis of HF, serum BNP above 100 pg/mL, any established cardiovascular disease, presence of retinopathy (at ophtalmology), peripheral artery disease, peripheral or cardiac neuropathy, respiratory insufficiency or diagnosis of chronic obstructive pulmonary disease (more than moderate airflow obstruction (forced expiratory volume in 1 s [FEV1] to forced vital capacity [FVC] ratio < 0.70 and FEV1 < 50% of predicted FEV1) and/or restrictive pattern (< 80% of predicted FVC)); pulmonary hypertension; any acute or chronic inflammatory disease; severe obesity defined as body mass index (BMI) > 40 kg/m^2^; uncontrolled blood pressure defined as BP > 160/100 mmHg; impaired kidney function defined as estimated glomerular filtration rate (eGFR) < 60 mL/min/1.73 m^2^; uncontrolled arrhythmias (including atrial fibrillation); any more than mild valvular disesase; inability to cycle due orthopedic limitations; poor echocardiographical acoustic windows; ongoing pregnancy or breastfeeding.

The definitive inclusion/exclusion criteria were reassessed both after the baseline evaluation and after the iCPET. The Local Ethic Committee approved the study protocol. All patients gave written informed consent before enrolment.

### Patient characterization

i. Clinical characterization
  A full clinical history was obtained. Baseline demographic data, anthropometric variables (height, weight and body mass index, BMI), functional status, cardiovascular risk factors (e.g. family history of cardiovascular disease, alcohol and smoking habits), comorbidities (e.g. arterial hypertension, dyslipidemia), and medication were also recorded. A thorough physical exam was also performed, including resting vital parameters. All patients underwent a resting cardiac echocardiography (see later for “baseline, speckle tracking, and exercise echocardiography”) and electrocardiogram.
ii. Biohumoral characterization
  Blood cell count, HbA_1c_, blood lipids, creatinine, electrolytes, uric acid, hepatic function, urinalysis, erythrocytes sedimentation rate, high-sensitivity C-reactive protein, BNP, urine albumin-to-creatinine ratio (ACR) were recorded at baseline. eGFR was calculated through the CKD-EPI formula.
iii. Vascular assessment
  Patients underwent peripheral vascular disease assessment through the cardio-ankle vascular index (CAVI) and ankle-brachial index (ABI) with the Vascular Screening System VaSera VS-1500 N® (Fukuda Denshi, Japan) to rule out peripheral vascular disease. Endothelial function was assessed by downstream hyperemic response to ischemia using an EndoPAT device (EndoPAT 2000, Itamar Medical Ltd., Caesarea, Israel), according to standard procedures [10]. The reactive hyperemia index (RHI) was calculated as the ratio between post- and pre-occlusion amplitudes of the pulse, normalized to the contralateral arm.
iv. Screening of neuropathy
  All patients underwent a thorough neurological clinical examination by a trained physician, as well as screening of neurological complications by the Semmens-Weinstein monofilament test and Neurotester® to exclude the presence of neurological complications. The monofilament test was performed according to standard procedures [11] for the screening of peripheral sensory-motor neuropathy. The screening of the cardiac autonomic neuropathy (CAN) was performed with the Neurotester® (Meteda srl, San Benedetto del Tronto, Italy): variations in RR intervals in response to the Valsalva manoeuvre, lying-to-standing, and deep breathing, according to standard procedures [12]. The three tests were repeated three times each for each patient. Two or more abnormal tests, based on age-related normal values, identified the presence of CAN.

### Cardiopulmonary exercise test (CPET) protocol

A symptom-limited graded ramp bicycle exercise test was performed in the semi-supine position on a tilting, dedicated, microprocessor-controlled stress echocardiography cycle ergometer (Ergoline ergoselect 2000 GmbH, Germany). A 12-lead electrocardiogram and non-invasive arterial saturation and blood pressure (BP) were monitored continuously. Heart rate (HR) and brachial BP were measured at rest and every minute during exercise using a validated automatic device (Omron M6 Comfort, Kyoto, Japan). The expected VO_2peak_, estimated on the bases of patient age, height, weight and clinical history [1], was used to adjust the ramp increments (Watt) to reach the patient’s estimated VO_2peak_ in 8 to 12 min. The protocol included two minutes of unloaded pedalling and four minutes of recovery after peak effort. Then, we excluded from the analysis patients who did not reach a RER > 1.0 during the exercise test. Breath-by-breath minute ventilation, carbon dioxide production (VCO_2_), and VO_2_ were measured using a dedicated cardiopulmonary diagnostic device (Blue Cherry, Geratherm Respiratory GmbH, Germany). We defined VO_2peak_ as the highest median value of the two 30-s intervals of the last minute of exercise, as previously extensively validated [13–17]. An automatic procedure determined anaerobic threshold (AT) based on the V-slope, ventilatory equivalents and end-tidal partial pressure methods; AT was verified visually and, if necessary, recalculated [1].

The chronotropic response was calculated as the change in HR from rest to peak exercise, divided by the difference between the age-predicted maximal HR and the resting HR (i.e. HR reserve). Chronotropic incompetence was defined as the failure to achieve ≥ 80% of the HR reserve during exercise [18]. In patients on β-blockers or calcium-channel blockers, chronotropic incompetence was defined as the failure to achieve 62% of HR reserve [18].

### Baseline, speckle tracking, and exercise stress echocardiography protocol

All patients underwent a comprehensive transthoracic echocardiography examination at rest (GE healthcare vivid e95, Milwaukee, WI, USA) according to the International Recommendations [19]. Data collected at each stage, that is at baseline, after 4 min, at the AT, and at peak effort, included: left ventricle (LV) and atrial (LA) volumes, stroke volume (SV), peak E-wave and A-wave velocities, tissue Doppler imaging (TDI)-derived S’ and e’ at the septal and lateral mitral annulus, tricuspid regurgitation velocity and systolic pulmonary artery pressure (sPAP), tricuspid annular plane systolic excursion (TAPSE); LV volumes and LVEF were calculated from the apical two- and four-chamber views using the modified Simpson’s rule. SV was calculated by multiplying the LV outflow tract area at rest by the LV outflow tract velocity–time integral measured by pulsed-wave Doppler during each activity level, as previously validated [20–23]. Cardiac output was calculated as the multiplication of SV and HR. The Δ(a-v)O_2_ was estimated indirectly with a validated and previously used approach by different groups using both our combined iCPET approach [24] and in a different setting with CPET and right heart catheterization [25]. Images were acquired concurrently with breath-by-breath gas exchange measurements at both baseline and peak of exercise. All measurements were reported as the average of three beats.

We measured global longitudinal strain (GLS) from the apical long-axis view and two- and four-chamber views, ensuring a frame rate > 50 Hz (GE healthcare EchoPAC BT 12). We reported the average values from the three apical views at rest and low-load effort, within the first 4 min of exercise. We excluded poorly tracked segments and patients were not analysed if more than one segment per view was deemed unacceptable. STE-derived measurements were reported as the average of three beats.

### Statistical analysis

Analyses were performed using JMP Pro software version 13.2.1 (SAS Institute, Cary, NC). Values are presented as mean ± SD, or as median and interquartile range (IQR), for variables with normal and non-normal distribution, respectively. Variables with a non-normal distribution at Kolmogorov–Smirnov test were logarithmically transformed for parametric analysis. Comparisons between groups were made by the Student t-test for unpaired data for continuous variables and by the chi-square test for categorical variables. All tests were conducted at a two-sided α level of 0.05.

In order to exclude drug-related chronotropic incompetence, we performed a sensitivity analysis after having excluded the subgroup taking beta-blockers, which confirmed the results of the analysis on the whole population (see Additional file 1: Table S4). Factors with ascertained or potential influence on Δ(a-v)O_2_ were selected for univariate linear regression analysis (age, sex, BMI, duration of diabetes, HbA_1c_, smoke, presence of hypertension, treatment with metformin, creatinine, ACR, PCR, hemoglobin, RHI, CAVI, ABI, MBP at peak, SVR at peak, CO at peak, LVEF at rest and at peak, ΔLVEF, GLS at rest and at 4 min, ΔGLS, S’ at rest and at peak, ΔS’, E/e’ peak, VE/VCO_2_ slope). In order to reduce overfitting, clinical, biohumoral, echocardiography, and CPET variables showing a p value < 0.100 at univariate analysis, were pooled into the multiple for multivariate analysis. Factors with ascertained or potential influence on VO_2peak_ age, sex, BMI, duration of diabetes, HbA_1c_, creatinine, eGFR, ACR, PCR,

hemoglobin, RHI, CAVI, HR at peak, chronotropic incompetence, MBP at peak, SVR at peak, CO at peak, LVEF at rest and at peak, ΔLVEF, GLS at rest and at 4 min, ΔGLS, S’ at rest and at peak, ΔS’, E/e’ peak, VE/VCO_2_ slope) were selected for univariate linear regressions. Shapiro–Wilk test was used for normal data, and Breusch-Pagan/Cook-Weisberg test was used for heteroskedasticity in the multiple regression models. A p value < 0.05 was considered statistically significant.

## Results

### Baseline characteristics of the study population

According to the inclusion and exclusion criteria, 114 consecutive patients were recruited for the study from December 2017 to July 2020; after baseline evaluation, 26 were subsequently excluded because of definitive exclusion criteria (10 for suboptimal ultrasound images during the exercise, 10 for incapacity of performing a maximal CPET due to discomfort, 4 for ECG signs suggestive of ischemia, 2 for evidence of autonomic neuropathy); the analysis was performed on 88 T2D subjects who met the definitive inclusion/exclusion criteria. We defined exercise intolerance as the incapacity of reaching a VO_2peak_ > 80% of predicted VO_2max_, which occurred in 48 subjects (55%). Baseline characteristics of the whole population and according to the presence or absence of effort intolerance are reported in Table 1.

**Table 1:** Baseline characteristics of the study population

	All patients (n = 88)	Effort Intolerance		p value
		Yes (n = 48)	No (n = 40)	
*Demographic and clinical data*				
Male gender (n, %)	71 (81%)	42 (88%)	29 (73%)	ns
Age (years)	63.8 ± 9.2	62.0 ± 9.4	65.9 ± 8.5	0.0446
BMI (kg/m^2^)	29.0 ± 4.7	29.8 ± 5.4	28.0 ± 3.6	ns
Systolic BP (mmHg)	135.9 ± 15.5	136.5 ± 16.0	135.1 ± 15.1	ns
Diastolic BP (mmHg)	83.1 ± 9.6	83.3 ± 9.7	82.9 ± 9.7	ns
Alcohol (n, %)	5 (6%)	3 (6%)	2 (5%)	ns
Smoke (n, %)	14 (16%)	10 (21%)	4 (10%)	ns
Hypertension (n, %)	67 (76%)	38 (79%)	30 (73%)	ns
Dyslipidemia (n, %)	66 (75%)	35 (73%)	31 (76%)	ns
Duration of diabetes (years)	9.3 ± 8.0	9.9 ± 7.7	8.6 ± 8.4	ns
*Therapy*				
ACEi/ARBs (n, %)	56 (63%)	30 (63%)	25 (63%)	ns
Beta-blockers (n, %)	23 (26%)	12 (25%)	11 (27%)	ns
Mineralocortic. Rec. Ant. (n, %)	3 (3%)	2 (4%)	1 (2%)	ns
Hydrochlorothyazide (n, %)	13 (15%)	6 (13%)	7 (17%)	ns
Furosemide (n, %)	3 (3%)	3 (6%)	0 (0%)	ns
Statin (n, %)	63 (71%)	32 (67%)	30 (76%)	ns
Metformin, n (%)	76 (85%)	41 (85%)	34 (85%)	ns
GLP1R-A (n, %)	2 (2%)	1 (2%)	1 (2%)	ns
DPP4i (n, %)	2 (2%)	2 (4%)	0 (0%)	ns
SGLT2i (n, %)	0 (0%)	0 (0%)	0 (0%)	ns
Insulin (n, %)	14 (16%)	10 (21%)	4 (10%)	ns
*Biohumoral data*				
HbA_1c_ (mmol/mol)	56.8 ± 10.4	56.9 ± 11.1	56.5 ± 9.7	ns
Cholesterol (mg/dL)	163.6 ± 18.4	157.6 ± 39.2	172.3 ± 35.4	ns
HDL-c (mg/dL)	49.5 ± 13.3	46.9 ± 11.2	53.2 ± 14.4	0.0283
LDL-c (mg/dL)	100.0 ± 32.9	94.6 ± 33.2	107.5 ± 31.1	ns
Ttriglycerides (mg/dL)	129.9 ± 57.5	137.3 ± 57.6	119.7 ± 56.6	ns
Hemoglobin (g/dL)	14.3 ± 1.3	14.2 ± 1.4	14.3 ± 1.1	ns
Creatinin (mg/dL)	0.89 ± 0.22	0.89 ± 0.20	0.90 ± 0.24	ns
eGFR (mL/min/1.73mq)	86.5 ± 15.8	89.5 ± 17.1	82.9 ± 13.6	0.0476
Uric acid (mg/dL)	5.39 ± 1.39	5.49 ± 1.61	5.22 ± 1.10	ns
ACR (mg/g)	6.0 (0.0 – 14.3)	5.8 (1.9 – 27.6)	6.1 (0.0 – 9.9)	ns
CRP (mg/dL)	0.278 ± 0.450	0.360 ± 0.590	0.193 ± 0.189	ns
BNP (pg/mL)	16 (10 – 33)	14 (10 – 37)	16 (10 – 29)	ns
*Vascular and endothelial data*				
RHI endoPAT	0.62 ± 0.28	0.63 ± 0.25	0.60 ± 0.32	ns
CAVI mean	9.32 ± 1.59	9.01 ± 1.69	9,67 ± 1.40	ns

The study population is reported as a whole and divided in two groups based on the achievement of a peak oxygen uptake > 80% of the maximal theorical oxygen uptake. P values were calculated with a student t-test and were reported as “ns” if non significant

The two groups had similar numerosity, sex prevalence, BMI, glycemic control, blood pressure values, prevalence of comorbidities, treatment for diabetes and cardio-active therapy. The group with preserved exercise capacity was slightly older, with a congruent marginally lower eGFR despite comparable values of serum creatinine. The group reaching a lower VO_2peak_ showed lower HDL-cholesterol values, despite comparable values of other blood lipids and prevalence of lipid lowering treatment, diuretics and beta blockers. At baseline echocardiography (Table 2), all patients showed normal RV and LV dimensions, mass, diastolic, and 2D systolic function (LVEF), with no difference between the two groups. Conversely, there was a difference in the TDI and speckle-tracking indices, so that the group achieving a lower VO_2peak_ showed a 10% lower baseline S’ and GLS values at rest.

**Table 2:** Results of cardiopulmonary exercise test and exercise echocardiography

	All patients (n = 88)	Effort Intolerance		p value	“Adjusted” p value
		Yes (n = 48)	No (n = 40)		
*Cardiopulmonary exercise test*					
Workload (W)	118 ± 30	113 ± 25	126 ± 34	0.0460	< 0.0001
Time of effort (min)	11.4 ± 2.0	10.8 ± 1.8	12.0 ± 2.0	0.0050	0.0008
HR rest (bpm)	79.9 ± 13.6	80.5 ± 12.9	79.3 ± 14.6	ns	ns
HR peak (bpm)	132.9 ± 18.6	129.2 ± 19.0	137.4 ± 17.2	0.0361	0.0015
HR peak (%max)	86.1 ± 12.0	82.6 ± 12.4	90.2 ± 10.2	0.0023	0.0015
HR reserve (bpm)	74.7 ± 15.7	76.1 ± 14.6	73.0 ± 16.9	ns	ns
Chronotr. incomp. (n, %)	46 (53%)	34 (71%)	12 (30%)	0.0001	0.0005
MBP rest (mmHg)	102.9 ± 10.3	103.3 ± 10.9	102.5 ± 9.6	ns	ns
MBP peak (mmHg)	146.5 ± 16.3	146.9 ± 18.2	146.1 ± 14.0	ns	ns
RER peak	1.08 ± 0.06	1.09 ± 0.07	1.07 ± 0.06	ns	ns
VO_2_/work slope	10.5 ± 1.5	9.9 ± 1.2	11.2 ± 1.4	< 0.0001	< 0.0001
VO_2_ rest (mL/min/kg)	4.1 ± 9.1	3.7 ± 1.2	4.6 ± 1.3	0.0015	0.0005
VO_2_ AT (mL/min/kg)	16.2 ± 4.9	13.7 ± 2.7	19.5 ± 5.2	< 0.0001	< 0.0001
VO_2_ AT (%peakVO_2_)	84.5 ± 11.9	81.5 ± 13.7	88.8 ± 7.7	0.0115	0.0047
VO_2_ peak (mL/min/kg)	18.8 ± 5.1	16.45 ± 3.19	21.69 ± 5.39	< 0.0001	< 0.0001
VO_2_ peak (%VO_2max_)	79.0 ± 17.0	66.7 ± 8.8	93.6 ± 11.9	< 0.0001	< 0.0001
VE/VCO_2_ slope	27.5 ± 3.8	27.2 ± 4.1	27.8 ± 3.3	ns	ns
VD/VT (%)	15.6 ± 4.1	16.0 ± 3.9	15.2 ± 4.4	ns	ns
O_2_ pulse peak (mL/bpm)	11.7 ± 2.8	11.3 ± 2.5	12.3 ± 3.0	ns	< 0.0001
O_2_ pulse peak (%max)	94.2 ± 18.0	84.3 ± 14.0	106.0 ± 15.0	< 0.0001	< 0.0001
AV O_2_ diff rest (mL/dL)	6.5 ± 2.3	6.1 ± 2.4	6.9 ± 2.3	ns	0.0218
AV O_2_ diff peak (mL/dL)	11.9 ± 3.2	11.3 ± 3.1	12.7 ± 3.3	0.0399	0.0002
*Exercise echocardiography*					
EDVi (mL/m^2^)	51.0 ± 11.1	51.7 ± 10.5	51.0 ± 13.0	ns	ns
LVMi (g/m^2^)	86.3 ± 16.6	86.6 ± 14.4	87.4 ± 20.8	ns	ns
LAVi (mL/m^2^)	24.7 ± 7.6	24.5 ± 8.2	25.5 ± 7.5	ns	ns
SV rest (mL)	69.1 ± 14.8	69.5 ± 13.3	68.6 ± 13.0	ns	ns
SV peak (mL)	102.1 ± 22.8	103.3 ± 26.6	100.7 ± 22.1	ns	ns
CO rest (L/min)	5.5 ± 1.2	5.5 ± 1.3	5.4 ± 1.2	ns	ns
CO peak (L/min)	13.7 ± 3.7	13.4 ± 3.7	14.0 ± 3.8	ns	ns
LVEF rest (%)	58.9 ± 4.3	58.6 ± 0.7	58.6 ± 1.0	ns	ns
LVEF peak (%)	67.6 ± 5.6	66.5 ± 6.1	68.9 ± 4.8	ns	ns
ΔEF	8.6 ± 3.7	8.0 ± 4.1	9.5 ± .31	ns	0.0412
Contractility reserve (n, %)	53 (60%)	23 (26%)	30 (34%)	0.0154	0.0386
GLS rest (%)	16.3 ± 2.6	15.7 ± 2.5	17.0 ± 2.6	0.0321	ns
GLS 4 min (%)	18.4 ± 3.0	17.5 ± 2.9	19.5 ± 2.8	0.0044	0.0048
ΔGLS	2.1 ± 1.5	1.7 ± 1.5	2.5 ± 1.8	0.0293	0.0093
S’ mean rest (cm/sec)	9.2 ± 1.8	8.8 ± 1.8	9.6 ± 1.7	0.0364	ns
S’ mean peak (cm/s)	14.3 ± 3.0	13.5 ± 2.8	15.2 ± 3.0	0.0085	0.0006
ΔS’ mean	5.1 ± 2.1	4.7 ± 1.9	5.6 ± 2.3	ns	0.0023
E/e’ rest (cm/s)	8.3 ± 2.2	8.6 ± 2.3	8.0 ± 2.2	ns	ns
E/e’ peak (cm/sec)	8.6 ± 2.0	8.7 ± 2.1	8.4 ± 1.9	ns	ns
SVR rest (dyne*s/cm)	1,583 ± 367	1,578 ± 383	1,588 ± 353	ns	ns
SVR peak (dyne*s/cm)	911 ± 218	932 ± 230	885 ± 203	ns	ns
TAPSE/sPAP peak	0.95 ± 0.23	0.96 ± 0.21	0.94 ± 0.26	ns	ns
TAPSE/CO peak	2.24 ± 0.62	2.33 ± 0.64	2.12 ± 0.58	ns	ns

The whole population and the two groups divided by the presence or absence of effort intolerance, are reported. P values were calculated as Student’s t test for comparisons of the means of the two groups, and “adjusted” p values were calculated after adjustment for age, sex, and BMI

### Cardiopulmonary exercise test

**Fig. 1:**
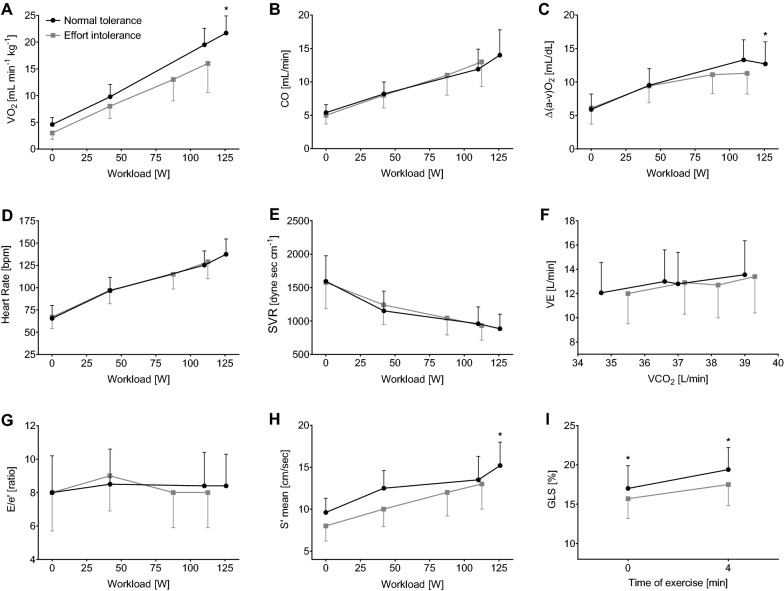
Graphic representation of nine key variables obtained during imaging-cardiopulmonary exercise test. Grey lines represent the group with effort intolerance, black lines represent the control group. Significant differences are highlighted by a star (*)

All patients performed a maximal exercise test, as defined by the maximal respiratory exchange ratio steadily greater than 1.05 at peak exercise according to guidelines [1]. The group with reduced exercise capacity achieved a 24% lower VO_2peak_ and a 10% lower peak workload and peak heart rate, while the mean systolic and diastolic blood pressure values were comparable throughout the test. We observed similar results at the sensitivity analysis to exclude drug-related chronotropic insufficiency, observing similar results (see Additional file 1: Table S4); however, these data should be interpreted considering the decrease in sample size. No difference was identified in ventilatory or gas exchange parameters, while the anaerobic threshold (AT) was reached earlier in the subjects with effort intolerance both in absolute terms, as well as when expressed as % of VO_2peak_. The group with reduced exercise capacity showed a higher prevalence of chronotropic incompetence, a lower VO_2_/workload slope, a reduced oxygen pulse at peak, as well as an impaired peripheral oxygen extraction [(a-v)ΔO_2_] at peak. These results are reported in Table 2 and in Fig. 1.

### Exercise echocardiography

During exercise, cardiac output (CO), LVEF, S’, GLS, and E/e’ all increased linearly with the workload. A reduced systolic reserve was observed in the group with effort intolerance in the form of: lower prevalence of subjects with a normal contractility reserve (*i.e.* an increase in LVEF > 7.5%), reduced ΔS’ (peak vs baseline), as well as in reduced early (4 min of exercise) GLS and GLS change from baseline (ΔGLS). LV diastolic indices, stroke volume, and cardiac output did not differ between the two groups throughout the iCPET test, as well as the right heart indices did not change throughout the test (TAPSE, sPAP, TAPSE/sPAP, TAPSE/CO) (Table 2 and Fig. 1).

### Regression analysis

**Fig. 2:**
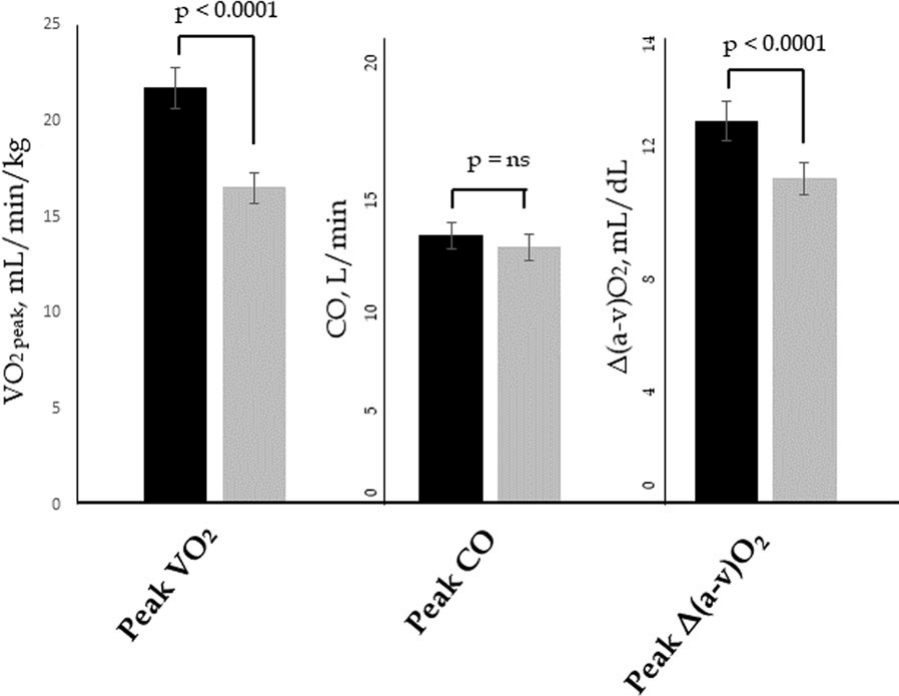
Linear regressions between systolic indices, peripheral oxygen extraction, and peak oxygen uptake. A significant positive linear correlation exists between S’ (**A**) at peak/change in S’ and VO_2peak_ (**B**), as well as between GLS at 4 min and VO_2peak_ (**C**) and change in GLS and VO_2peak_ (**D**). A positive linear correlation was also observed between Δ(a-v)O_2_ and GLS at 4 min (**E**) and change in GLS (**F**)

According to the Fick’s equation, whole-body oxygen uptake is determined by CO and $\Delta (\mathrm{a}-\mathrm{v}){\mathrm{O}}_{2}$; in our study, peripheral oxygen extraction explains the impaired exercise capacity (Fig. 2). Therefore, we focused the regression analysis on Δ(a-v)O_2_. In the whole population, univariate determinants of Δ(a-v)O_2_ at peak were: sex, hemoglobin levels, peak CO, peak systemic vascular resistance, peak mean arterial pressure, GLS at 4 min and ΔGLS. In a multiple regression model, only male sex (st-ß 0.35, p = 0.0002), hemoglobin levels (st-ß 0.22, p = 0.0133), and ΔGLS (st-ß 0.21, p = 0.0486) were independent predictors of peak Δ(a-v)O_2_ (see Table 3).

**Table 3:** Determinants of peripheral oxygen extraction

	Univariate		Multivariate	
Variable	st-β	p	st-β	p
Age	0.01	ns		
Sex	0.07	0.0138	0.35	0.0002
BMI	0.01	ns		
Duration of diabetes	− 0.01	ns		
HbA_1c_	− 0.04	ns		
Smoke	0.02	ns		
Hypertension	0.02	ns		
metformin	− 0.01	ns		
Creatinine	0.02	ns		
ACR (log)	− 1.02	ns		
PCR (log)	− 0.50	ns		
Hemoglobin	0.70	0.0103	0.22	0.0133
RHI endoPAT	− 0.06	ns		
CAVI	0.20	ns		
ABI	0.08	ns		
MBP peak	− 0.05	0.0175	− 0.02	ns
SVR peak	0.01	< 0.0001	0.01	ns
CO peak	− 0.52	< 0.0001	− 0.30	ns
LVEF rest	− 0.01	ns		
LVEF peak	0.01	ns		
Δ LVEF	0.10	ns		
GLS rest	0.12	ns		
GLS 4 min	0.34	0.0060	0.10	ns
Δ GLS	0.19	< 0.0001	0.21	0.0486
S’ rest	0.06	ns		
S’ peak	0.06	ns		
Δ S’	0.16	ns		
E/e’ peak	0.12	ns		
VE/VCO_2_ slope	− 0.06	ns		

**Fig. 3:**
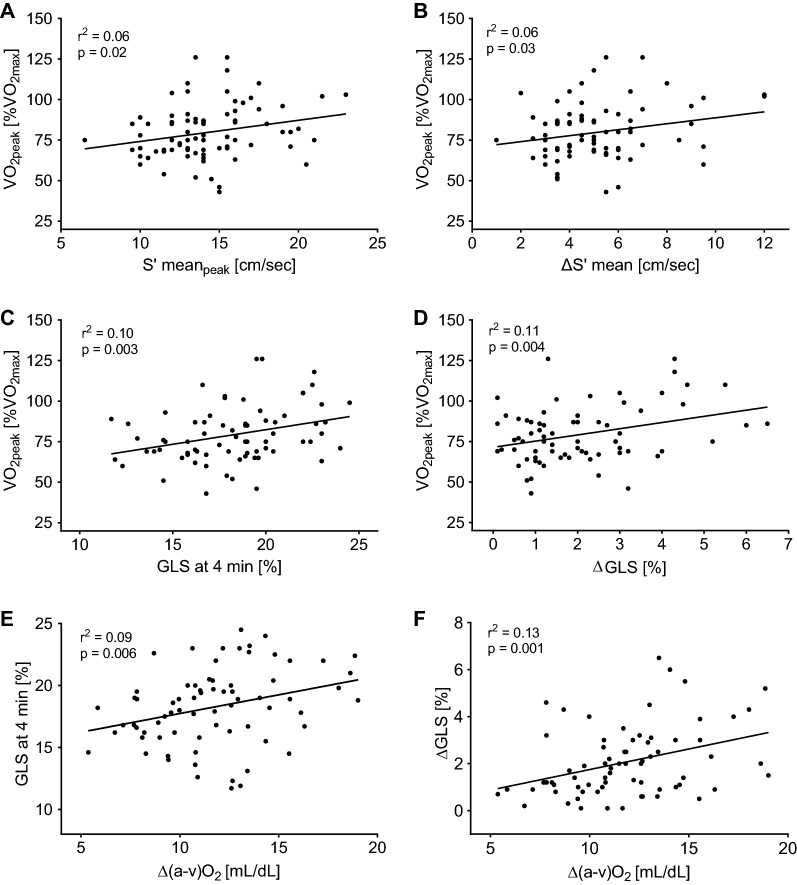
Determinants of peak oxygen uptake according to Fick’s principle. VO_2_ is determined by cardiac output (CO) and peripheral oxygen extraction (Δ(a-v)O_2_). The p value for the difference between the two study groups is shown on top of the diagrams

To gain insight on the link between reduced peak oxygen utilization and systolic dysfunction, linear regression analyses were performed on the two corresponding sets of variables in the whole population (Fig. 3). Significant positive linear correlations were found between S’ at peak and VO_2peak_ (Panel A), as well as between ΔS’ and VO_2peak_ (panel B), between GLS at 4 min and VO_2peak_ (panel C) and ΔGLS and VO_2peak_ (panel D). Also, we observed a significant correlations between Δ(a-v)O_2_ and GLS at 4 min (panel E) and change in GLS (Panel F).

## Discussion

### Effort intolerance in type 2 diabetes

We examined 88 older adults with well-controlled and uncomplicated T2D undergoing a maximal iCPET. The observed value of VO_2peak_ in the whole population (18 mL/kg/min) falls far below the reference values for the general population of this age group (women 31 mL/kg/min; men 39 mL/kg/min) [26], confirming previous reports indicating poor exercise capacity in T2D subjects [4]. An identical mean value of VO_2peak_ (18.0 ± 6.6 mL/min/kg) has been recently reported in a larger and even younger (by 10 years) cohort of 224 asymptomatic subjects with T2D, falling into the lower 10% of the age-matched male general population distribution and the lower 20% of female general population [27]. Exercise intolerance, defined by VO_2peak_ below 80% of the predicted value according to Wasserman equation, is widely used to define negative prognosis in subjects suffering from heart disease [1]; still, it was present in most (55%) of our study population despite the absence of either vascular and autonomic diabetic complications, criteria for a definite diagnosis of HF, or any detectable significant cardiac impairment at resting assessment. The prevalence of this severe condition in uncomplicated T2D was not reported in previous studies, wherein the severity of effort intolerance has probably been underestimated. Indeed, the subjects in our cohort with effort intolerance show VO_2peak_ values that are commonly found in patients with overt HF, a population wherein such a reduced VO_2peak_ portends a rather poor prognosis [28].

The study population was homogeneous in demographic parameters, glycemic control, duration of diabetes, cardio-active and glucose-lowering therapy, vascular and endothelial function parameters, as well as the prevalence of comorbidities (Table 1). It is thus very difficult to predict effort intolerance based on the resting clinical phenotype alone. The difference in HDL cholesterol was small and seemingly driven by the slightly higher prevalence of male and overweight subjects in the group with low VO_2peak_. This, together with the presence of a small difference in age, prompted the decision to verify the differences in iCPET data after adjusting for age, sex and BMI (Table 2). Since effort intolerance is the hallmark of HF irrespective of LVEF [2], and that cardiorespiratory fitness is known to be a strong predictor of incident HFpEF in the T2D population [6], we sought to determine the associated alterations and mechanisms underpinning the reduced VO_2peak_ in T2D patients to gain insight on the earliest defects at the bases of the their higher HF vulnerability. Previous findings reported early development of fatigue in T2D as a perceived limitation of force-generating capacity that requires higher intensity of effort that might eventually reduce the exercise duration, and that can be highlighted by an early appearance of exhaustion during exercise and in higher fatigue with respect to controls at any given workload, even when adjusted for the reduced VO_2peak_ [29, 30]. Still, the reasons for the decreased exercise tolerance are far from being clear, possibly encompassing any combination of myocardiogenic, skeletal myogenic, vasculogenic, or neurogenic determinants [4]; we sought to determining the associated alterations in the different organs and systems.

### Mechanisms of effort intolerance in type 2 diabetes

Our first finding is that effort intolerance is not due to a defect in mechanical efficiency (as was suggested for obese individuals [31, 32]), given that the slope of VO_2_ vs work-rate is steeper in subjects with preserved exercise tolerance (Fig. 1) and that we can exclude an impairment in ventilatory parameters. A reduced O_2_ supply could be related to central (lung and/or heart) or peripheral (hematologic, vascular, or mitochondrial) impairment [4]. We excluded lung disease, as all the patients underwent spirometry before exercise, whilst hematologic diseases were excluded after analysis of blood exams before enrollment. Then, imaging-CPET provides the opportunity to dissect the different components of the Fick’s equation, thanks to the possibility of measuring stroke volume with the simultaneous echocardiographic assessment. According to the Fick’s principle, a reduced peripheral oxygen extraction explains the impaired cardiopulmonary function in our population (Fig. 3). Previous studies have reported either a reduced or a normal peripheral extraction in T2D [33, 34]. Notably, both the study by Baldi et al. [33] and the more recent one by Kobayashi [35] performed in a similar population of asymptomatic T2D patients confirm our findings of a normal cardiac output with a Δ(a-v)O_2_ that was reduced by 20% compared to the control group and that correlated with the reduced VO_2peak_. Notably, in line with our findings, the Authors conclude that a reduced peripheral oxygen extraction might be regarded as a limitation to whole-body oxygen uptake. The older study showing normal Δ(a-v)O_2_ was conducted in a very small group of female adolescents [34], thus with poor clinical applicability.

The two groups of our study did not differ in biohumoral values, endothelial function, indices of pulmonary function, diastolic function indices, right heart indices, mean arterial pressure, and systemic vascular resistances throughout the entire iCPET. The subjects with effort intolerance showed higher prevalence of chronotropic incompetence. The large difference, however, is driven by the fact the patients of our study group fall close to the 80% threshold (mean HR peak%: 86.1%) thus a small difference in peak HR generates a major recruitment of subject with a diagnosis of chronotropic incompetence. In quantitative terms, the difference between the groups was small; subjects with effort intolerance exploited their HR reserve only 8% less than the others (82.6 vs 90.2%) with a difference in peak HR of just 8 beats/minute (129 vs 137 bpm). If we also consider that no subject had evidence of CAN at conventional tests and, more importantly, that HR kinetics and peak CO were superimposable in the two groups throughout the whole iCPET, it is unlikely that chronotropic incompetence is the cause of effort intolerance in our patient; it might rather be the consequence of their lower fitness.

Whilst crude indices of systolic performance such as SV, CO and LVEF were not different between the two study groups, less load-dependent indices (S’ and GLS) showed a gradient that was evident both in resting conditions and in exercise-induced changes. It is widely known that a reduced baseline GLS is an early marker of LV subclinical systolic dysfunction, being present both in HFpEF patients irrespective of the diabetic state [24] and in T2D subjects without HF [36, 37]—where it also predicts incident HF [38]. Our findings confirm most previous reports (although not all [39]) describing reduced S’ velocity of the mitral annulus measured through tissue Doppler in patients with T2D during exercise [40–42], an observation that was also related to myocardial fibrosis as measured though cardiac magnetic resonance [43]. Given the large prevalence of diastolic dysfunction in T2D subjects [38] and the results of a recent report by Gulsin et al. [44], we were surprised not to see alteration in E/e’ in our population with effort intolerance, neither at rest nor during exercise. In the work of Gulsin and coll., however, diastolic indices were not measured during exercise, and the association was essentially driven by a minority of subjects with a baseline E/e’ > of 12.5, and in the whole population the effect size was small with + 1 units of E/e’ justifying -0.3 ml/kg/min of VO_2peak_. Based on our data, diastolic dysfunction is not relevant for explaining effort intolerance in T2D.

### Determinants of peripheral oxygen extraction

In multivariate analysis, the determinants of Δ(a-v)O_2_—the main factor explaining the reduced VO_2peak_ in our population—were sex, hemoglobin levels, and ΔGLS (Table 3). This confirms previous observations on the sex-related differences in effort tolerance and possibly the different risk of HF development seen between T2D males and females [45]. It also supports the deterministic role of hemoglobin in ambient oxygen availability [1]. At rest, diabetic individuals show reduced ATP release from red blood cells in response to hemoglobin desaturation activated through endothelial purinergic receptors that trigger nitric oxide-dependent and independent arteriolar vasodilation, and that significantly impacts on muscle blood flow [46]; however, its relevance during exercise in unknown. Interestingly, SGLT-2 inhibitors, which protect T2D patients from HF incidence and decompensation through unknown mechanisms, significantly rise hemoglobin as a side effect [47]. Since SGLT-2 inhibitors have been recently demonstrated to ameliorate aerobic fitness in T2D subjects both without HF [48] and with HFpEF [49], one can speculate that the hemoconcentration with increased hemoglobin obtained with their pharmacological effect might increase Δ(a-v)O_2_ and partly explain the increased whole-body oxygen uptake. Finally, the strength of the correlation between subclinical systolic dysfunction to peak Δ(a-v)O_2_ and VO_2peak_ is a novel finding suggesting a strong link between skeletal and cardiac muscle pathology in T2D. This relationship was previously demonstrated in animal models of T2D [50], while in humans previous studies have reported either a reduced GLS or S’ at rest [51], reduced longitudinal systolic reserve [40], or reduced Δ(a-v)O_2_ in T2D subjects with effort intolerance [33], but none has reported a direct relationship between peripheral oxygen extraction and systolic indices, neither at rest nor during exercise.

### Clinical value

Taken together, the results of the present study suggest a myogenic limitation of whole-body oxygen uptake in T2D limiting exercise tolerance, with a tight interplay between myocardial and skeletal muscles. Whether this is secondary to a reduced number of mitochondria, a mitochondrial functional impairment, altered myofibrillar structure and/or composition, muscle microvasculature, or to systemic regulators of muscle perfusion [4] goes beyond the purpose of this study. However, the lack of relationships with total peripheral resistances and endothelial function supports a primitive muscle cell impairment involving both skeletal and myocardial muscle. The lower anaerobic threshold, also when expressed in terms of % VO_2peak_, indicates a reduced aerobic capacity and strongly supports the hypothesis of a mitochondrial defect either in number or in function, as previously observed in this population [52, 53]. The observation that exercise training can increase whole-body oxygen consumption through an amelioration of skeletal muscle energetics further sustains this point [54, 55].

The combination of reduced VO_2peak,_ Δ(a-v)O_2_, and GLS of the subjects with T2D and effort intolerance observed in the present study represents a phenotype which is also shared by the patients with HFpEF wherein reduced peripheral oxygen extraction and/or GLS appear as the major determinants of effort intolerance [37, 56, 57]. Being each trait less pronounced in our population, we speculate that this condition might represent an intermediate phenotype and—if eventually confirmed to be so prevalent in T2D—might explain the excess prevalence of HFpEF among these patients [2, 56]. Of note, our population did not show diastolic dysfunction as a key determinant of effort intolerance, at least not as importantly as in HFpEF subjects [2], probably marking a further step forward towards overt HFpEF. Due to the “diabesity” pandemic and the high incidence (and costs) of HFpEF, new strategies for the early identification of the patients at risk of HFpEF are needed. In this context, the iCPET might reveal a useful screening tool [58]. Longitudinal trials evaluating the transition from T2D with effort intolerance to overt HFpEF would provide support to our hypothesis, as well as clinical intervention trials aiming at verifying whether ameliorating peripheral oxygen extraction (*e.g.* exercise) is possible to prevent the development of HFpEF in T2D patients.

## Concluding remarks

Effort intolerance is severe and highly prevalent in uncomplicated, otherwise asymptomatic T2D, and is mainly driven by a primitive muscular impairment involving both skeletal and myocardial muscle in the form of impaired peripheral oxygen extraction and a reduced systolic reserve, despite preserved LVEF and cardiac output. These alterations closely resemble the major clinical features of HFpEF and could represent an intermediate pathological condition.

### Strengths and limitations

One strength of the present study is the sample size (greater than previous reports), the multi-parametric analysis performed (previous works were focused on specific organ dysfunctions, or did not use exercise echocardiography, or considered resting variables), the careful exclusion of micro- and macrovascular complications (previous works frequently included complicated patients and highlighted the contribution of specific complications to reduced aerobic capacity), and the study of effort intolerance within the diabetic population (whilst previous works were focused on differences between diabetic and non-diabetic subjects, and the exact prevalence and mechanisms of effort intolerance within the T2D population is unknown).

We recognize some limitations of the present work. This is a single-centre, cross-sectional study with a relatively small sample size. We only focused on asymptomatic, uncomplicated T2D; therefore, the results should not be applied to different cohorts. We acknowledge that the technical challenge of acquiring echocardiography images during exercise may affect SV and CO measurements, despite the technique has been extensively validated and used by different groups [24]. Also Δ(a-v)O_2_ was not directly measured, however our method has been extensively validated, used by several investigators [24, 25]; and, most importantly, our values are in line with observations reporting both non-invasive and invasive oxygen extraction data [57, 59]. Our imaging protocol was performed in a semi-supine position for a better echocardiographic evaluation [13]; caution is advised to extend our results to other types of exercise (supine or upright).

## Supplementary Information

**Additional file 1: Table S4.** Sensitivity analysis performed after having excluded all subjects taking beta-blockers. And repeating the statistical analysis as described for the whole population.

## Data Availability

Not applicable.

## Abbreviations

AT: Anaerobic threshold
BMI: Body mass index
CO: Cardiac output
CPET: Cardiopulmonary exercise test
SV: Stroke volume
E’: Early left ventricular filling phase velocity
HF: Heart failure
HFpEF: Heart failure with preserved ejection fraction
HR: Heart rate
METs: Metabolic equivalents
PaCO_2_: Arterial partial pressure of carbon dioxide
PETCO_2_: End-tidal carbon dioxide partial pressure
S’: Peak systolic annular velocity
T2D: Type 2 diabetes mellitus
VCO_2_: Carbon dioxide emission measured at the mouth
V_D_/V_T_: Physiological dead space/tidal volume ratio
VE: Pulmonary minute ventilation
VE/VCO_2_: Ventilatory equivalent for carbon dioxide or “ventilatory efficiency”
VO_2_: Oxygen uptake measured at the mouth
VO_2_/HR: Oxygen pulse
VO_2max_: Maximal theorical oxygen uptake
VO_2peak_: Peak oxygen uptake
Δ(a-v)O_2_: Peripheral oxygen extraction
