# Supplementary material for: Mechanisms of reduced peak oxygen consumption in subjects with uncomplicated type 2 diabetes
Source: Cardiovasc Diabetol. 2021 Jun 22;20:124. doi: 10.1186/s12933-021-01314-6 (PMC8218418; doi:10.1186/s12933-021-01314-6)
Supplement: Supplementary file 1 — Additional file 1: Table S4. Sensitivity analysis performed after having excluded all subjects taking beta-blockers. And repeating the statistical analysis as described for the whole population. [file 12933_2021_1314_MOESM1_ESM.docx]

Table S4

|  | Effort Intolerance | | | p value |
| --- | --- | --- | --- | --- |
|  | Yes  (n = 44) | | No  (n = 30) |  |
| Workload (W) | 112.5 ± 24.8 | 128.5 ± 35.3 | | 0.0242 |
| *Time of effort (min)* | 10.9 ± 1.9 | 12.3 ± 2.1 | | 0.0035 |
| *HR rest (bpm)* | 81.9 ± 12.3 | 79.4 ± 14.2 | | 0.4364 |
| *HR 4 min (bpm)* | 98.9 ± 2.2 | 97.3 ± 12.5 | | 0.6390 |
| *HR at AT (bpm)* | 118.0 ± 17.0 | 128.3 ± 12.4 | | 0.0085 |
| *HR peak (bpm)* | 131.0 ± 18.6 | 140.8 ± 15.1 | | 0.0193 |
| *HR peak (%max)* | 83.6 ± 12.4 | 92.0 ± 9.1 | | 0.0024 |
| *Chronotr. response (bpm)* | 68.1 ± 24.2 | 84.1 ± 18.2 | | 0.0031 |
| *Chronotr. Incomp. (n, %)* | 30 (68%) | 9 (30%) | | 0.0019 |
| *MBP rest (mmHg)* | 103.2 ± 11.4 | 102.6 ± 9.9 | | 0.8065 |
| *MBP peak (mmHg)* | 146.6 ± 18.6 | 147.8 ± 11.8 | | 0.7459 |
| *RER peak* | 1.09 ± 0.06 | 1.08 ± 0.06 | | 0.1098 |
| *VO_2_/work slope* | 9.8 ± 1.2 | 11.2 ± 1.1 | | <0.0001 |
| *VO_2_ rest (mL/min/kg)* | 3.8 ± 1.2 | 4.65± 1.3 | | 0.0189 |
| *VO_2_ at 4 min (mL/min/kg)* | 8.5 ± 1.7 | 9.9 ± 2.3 | | 0.0075 |
| *VO_2_ AT (mL/min/kg)* | 13.7 ± 2.8 | 20.5 ± 5.2 | | <0.0001 |
| *VO_2_ AT (%peakVO_2_)* | 84.2 ± 7.7 | 88.8 ± 7.7 | | 0.0198 |
| *VO_2_ peak (mL/min/kg)* | 16.5 ± 3.2 | 21.7 ± 5.4 | | <0.0001 |
| *VO_2_ peak (%VO_2max_)* | 66.0 ± 8.7 | 95.7 ± 12.4 | | <0.0001 |
| *VE/VCO_2_ slope* | 27.6 ± 4.2 | 27.6 ± 3.4 | | 0.6870 |
| *VD/VT (%)* | 15.8 ± 4.0 | 14.9 ± 4.5 | | 0.4046 |
| *O_2_ pulse peak (mL/bpm)* | 11.0 ± 2.5 | 12.4 ± 2.9 | | 0.0293 |
| *O_2_ pulse peak (%max)* | 82.4 ± 12.9 | 106.3 ± 14.9 | | <0.0001 |
| *AV O_2_ diff rest (mL/dL)* | 6.0 ± 2.2 | 6.6 ± 2.0 | | 0.2308 |
| *AV O2 diff 4 min (mL/dL)* | 9.2 ± 2.3 | 9.5 ± 2.4 | | 0.6267 |
| *AV O2 diff AT (mL/min)* | 11.1 ± 2.9 | 13.5 ± 3.5 | | 0.0043 |
| *AV O_2_ diff peak (mL/dL)* | 11.2 ± 3.1 | 12.8 ± 3.2 | | 0.0369 |
| *SV rest (mL)* | 69.1 ± 15.7 | 68.2 ± 11.1 | | 0.7939 |
| *SV peak (mL)* | 101.7 ± 23.2 | 101.8 ± 22.6 | | 0.9882 |
| *CO rest (L/min)* | 5.6 ± 1.3 | 5.3 ± 0.9 | | 0.2091 |
| *CO at 4 min (L/min)* | 8.3 ± 2.0 | 8.2 ± 1.4 | | 0.7695 |
| *CO at AT (L/min)* | 11.3 ± 3.0 | 12.3 ± 3.0 | | 0.1963 |
| *CO peak (L/min)* | 13.4 ± 3.8 | 14.4 ± 3.8 | | 0.2792 |
| *LVEF rest (%)* | 58.5 ± 4.7 | 59.7 ± 3.6 | | 0.2865 |
| *LVEF peak (%)* | 66.2 ± 6.3 | 69.0 ± 4.7 | | 0.0425 |
| *ΔEF* | 7.7 ± 4.0 | 9.4 ± 3.4 | | 0.0584 |
| *Contractility reserve (n, %)* | 20 (45%) | 22 (73%) | | 0.0396 |
| *GLS rest (%)* | 15.7 ± 2.6 | 17.2 ± 2.4 | | 0.0214 |
| *GLS 4 min (%)* | 17.4 ± 3.0 | 19.7 ± 2.7 | | 0.0036 |
| *ΔGLS* | 1.7 ± 1.2 | 2.5 ± 1.6 | | 0.0279 |
| *S’ mean rest (cm/sec)* | 8.9 ± 1.7 | 9.9 ± 1.8 | | 0.0231 |
| *S’ at 4 min (cm/sec)* | 11.0 ± 2.2 | 12.0 ± 1.8 | | 0.0557 |
| *S’ at AT (cm/sec)* | 12.7 ± 2.5 | 14.1 ± 2.1 | | 0.0158 |
| *S’ mean peak (cm/sec)* | 13.5 ± 2.9 | 15.6 ± 3.1 | | 0.0051 |
| *ΔS’ mean* | 4.6 ± 1.8 | 5.7 ± 2.4 | | 0.0373 |
| *E/e’ rest (cm/sec)* | 8.6 ± 2.4 | 7.5 ± 1.8 | | 0.0556 |
| *E/e’ peak (cm/sec)* | 8.7 ± 2.1 | 8.6 ± 1.7 | | 0.8489 |
| *SVR rest (dyne*s/cm)* | 1,550 ± 371 | 1,597 ± 319 | | 0.5856 |
| *SVR peak (dyne*s/cm)* | 927 ± 228 | 872 ± 209 | | 0.3057 |
